# Clinical Outcomes in Blood Culture-Positive Versus Blood Culture-Negative Infective Endocarditis: A Systematic Review and Meta-Analysis

**DOI:** 10.7759/cureus.88134

**Published:** 2025-07-17

**Authors:** Huzaifa Rehman, Anurag Rawat, Fahad Shaukat Gill, Arti Kukreja, Yuliia Oliinyk, Sandipkumar S Chaudhari, Mohammed Qasim Rauf, Areeba Khan

**Affiliations:** 1 General Medicine, Avicenna Medical College, Lahore, PAK; 2 Opthalmology, Rochdale Infirmary Hospital, Rochdale, GBR; 3 Interventional Cardiology, Himalayan Institute of Medical Sciences, Dehradun, IND; 4 Medicine, Shalamar Medical and Dental College, Lahore, PAK; 5 Medicine, Ghulam Muhammad Mahar Medical College, Sukkur, PAK; 6 Medicine, Odesa National Medical University, Odesa, UKR; 7 Cardiothoracic Surgery, University of Alabama at Birmingham, Birmingham, USA; 8 Family Medicine, University of North Dakota School of Medicine and Health Sciences, Fargo, USA; 9 Orthopaedic Surgery, Hillingdon Hospital, London, GBR; 10 Critical Care Medicine, United Medical and Dental College, Karachi, PAK

**Keywords:** blood culture-negative, clinical outcomes, embolic events, infective endocarditis, meta-analysis

## Abstract

Infective endocarditis (IE) cases where blood cultures fail to identify causative organisms, known as blood culture-negative endocarditis (BCNE), represent a significant portion of all endocarditis diagnoses. This absence of microbiological identification creates therapeutic challenges, as clinicians cannot tailor antimicrobial therapy to specific pathogens. The relationship between microbiological culture results and patient prognosis continues to be an area requiring further investigation. This systematic review and meta-analysis compared clinical outcomes between blood culture-positive endocarditis (BCPE) and BCNE patients. We conducted a comprehensive search of PubMed/MEDLINE, Embase, Scopus, Web of Science, and Cochrane Library from inception to May 2025. Observational studies comparing outcomes between BCPE and BCNE in adult patients were included. Primary outcomes were mortality, embolic events, and need for surgery. Risk ratios (RRs) with 95% confidence intervals (CIs) were calculated using random-effects models. Seven studies encompassing 5,349 participants were included, with a pooled BCNE prevalence of 16.8%. No significant difference was found in mortality between BCPE and BCNE groups (RR: 0.98, 95% CI: 0.76-1.25). However, BCPE patients had a significantly higher risk of embolic events compared to BCNE patients (RR: 1.38, 95% CI: 1.16-1.63). No significant difference was observed in surgical intervention rates (RR: 0.98, 95% CI: 0.75-1.27). Secondary analysis revealed that BCPE patients had higher rates of fever and abscess formation but a lower incidence of heart failure compared to BCNE patients. While mortality and surgical outcomes were similar between groups, blood culture-positive IE patients demonstrated significantly higher embolic complications. These findings suggest that culture-positive status may serve as a marker for increased embolic risk, warranting enhanced monitoring and early intervention strategies in clinical practice.

## Introduction and background

Infective endocarditis (IE) remains a life-threatening condition with mortality rates exceeding 20%, despite advancements in diagnostic and therapeutic strategies [1]. A critical challenge in its management stems from blood culture-negative endocarditis (BCNE), which accounts for up to 30% of IE cases and complicates pathogen-directed treatment [1]. Historically, BCNE has been associated with delayed diagnosis, broader empirical antibiotic use, and potentially worse clinical outcomes compared to blood culture-positive endocarditis cases (BCPE) [2,3]. 

BCNE accounts for approximately 2.5% to 31% of all IE cases, with this variability largely attributed to prior antibiotic exposure, fastidious or atypical organisms, and limitations in laboratory detection [4]. Common pathogens missed by conventional blood cultures include *Coxiella burnetii*, *Bartonella* spp., and fungi, among others. In contrast, BCPE is generally associated with more straightforward pathogen identification, allowing for targeted antimicrobial therapy [5]. This distinction in microbiological diagnosis can significantly influence clinical decision-making, therapeutic planning, and prognosis. 

The absence of an identifiable pathogen in BCNE complicates treatment, often necessitating empiric broad-spectrum antimicrobial regimens, which may be suboptimal or unnecessarily toxic [6]. Furthermore, delayed or inappropriate therapy due to diagnostic uncertainty may contribute to worse clinical outcomes in BCNE patients [7]. Some studies have suggested that BCNE is associated with higher rates of complications, longer hospital stays, and increased mortality, while others report no significant difference in outcomes compared to BCPE [8,9]. This inconsistency in the literature highlights the need for a comprehensive synthesis of available evidence to guide clinicians in managing these complex cases. 

Understanding whether the presence or absence of positive blood cultures in IE influences outcomes, such as in-hospital mortality, embolic events, and need for surgical intervention, is required. A meta-analytical approach offers the opportunity to aggregate and analyze data across studies to assess these outcome differences systematically and quantitatively. Several meta-analyses compared the clinical outcomes between culture-positive and culture-negative subjects in different conditions, including sepsis and septic shock [10,11]. No meta-analysis has been conducted to compare the outcomes between culture-negative and culture-positive endocarditis subjects. The primary objective of this systematic review and meta-analysis is to compare clinical outcomes between blood culture-positive (BCPIE) and blood culture-negative IE (BCNIE) patients. 

## Review

Methodology 

Study Design

This meta-analysis was conducted in accordance with the Preferred Reporting Items for Systematic Reviews and Meta-Analyses (PRISMA) guidelines to ensure methodological rigor and transparency. 

*Literature Search* 

A comprehensive and systematic literature search was conducted to identify all relevant studies comparing outcomes between BCPE and BCNE. The search was designed in accordance with the PRISMA guidelines and performed across multiple electronic databases, including PubMed/MEDLINE, Embase, Scopus, Web of Science, and the Cochrane Library, from their inception of databases to 25 May 2025. The search strategy used a combination of Medical Subject Headings (MeSH), Emtree terms, and free-text keywords to maximize sensitivity and specificity. The following terms and their variants were used and combined with Boolean operators (AND, OR). The search strategy combined terms related to the condition of interest, specifically infective endocarditis, IE, and endocarditis, with terms describing the exposure groups, including blood culture-negative, culture negative, BCNE, blood culture-positive, culture positive, and BCPE. Outcome-related search terms encompassed broad clinical endpoints such as outcome, mortality, survival, prognosis, complications, clinical outcome, and treatment outcome, as well as specific mortality timeframes including hospital mortality, in-hospital mortality, 30-day mortality, and one-year mortality. Additional terms captured therapeutic interventions and their outcomes, specifically surgery, surgical outcome, and medical management. The search also incorporated terms related to prognostic assessment, including risk factors, predictors, and prognostic factors, to ensure comprehensive identification of relevant studies that examined determinants of outcomes in both BCPE and BCNE populations. Reference lists of included studies and relevant review articles were manually searched to identify additional eligible studies not captured by the electronic search strategy. 

*Eligibility Criteria* 

We included studies that met the following criteria: (1) observational studies (cohort, case-control, or cross-sectional) comparing outcomes between BCPIE and BCNIE; (2) studies that reported at least one of the following outcomes: all-cause mortality (in-hospital or follow-up), embolic events, and need for surgical intervention, (3) studies involving adult patients (≥18 years) diagnosed with definite or possible IE based. We excluded case reports, case series, editorials, reviews, and studies lacking comparative outcome data between BCPIE and BCNIE groups. 

All studies were imported into EndNote Software (Clarivate, Philadelphia, PA). Two independent reviewers (FG and AK) screened the titles and abstracts of all records retrieved through the database search. Articles that met the eligibility criteria or required further evaluation underwent full-text review. Disagreements between the reviewers regarding study inclusion were resolved through discussion and consensus. A third reviewer was consulted when disagreements persisted. Full-text articles were assessed to ensure they met all predefined inclusion criteria. For studies with overlapping populations or duplicate data, the most comprehensive or recent publication was included. 

Data Extraction 

Data extraction from the eligible studies was carried out independently by two reviewers using a pre-designed standardized form. The extracted details included the first author’s name, publication year, study type, geographic location, total sample size, characteristics of the study population, and reported outcomes. 

To evaluate the risk of bias in the included observational studies, two reviewers independently applied the Newcastle-Ottawa Scale (NOS). This tool assesses methodological quality across three key domains: selection of participants, comparability between groups, and outcome assessment. Based on the NOS scoring system, studies receiving 7 to 9 points were categorized as having a low risk of bias, scores of 4 to 6 indicated a moderate risk, and scores of 3 or below reflected a high risk of bias. 

Statistical Analysis 

All statistical analyses were performed using RevMan software (Cochrane, London, UK). For binary outcomes, risk ratios (RRs) along with 95% confidence intervals (CIs) were estimated using a random-effects model to address variability across studies. Statistical significance was defined as a p-value less than 0.05. Between-study heterogeneity was evaluated using the I² statistic, where values of 25%, 50%, and 75% indicated low, moderate, and high heterogeneity, respectively. Additionally, a p-value below 0.10 from the Cochran’s Q test was interpreted as evidence of significant heterogeneity. Sensitivity analyses were conducted by removing studies deemed to have a high risk of bias or those identified as outliers. 

Results 

**Figure 1:**
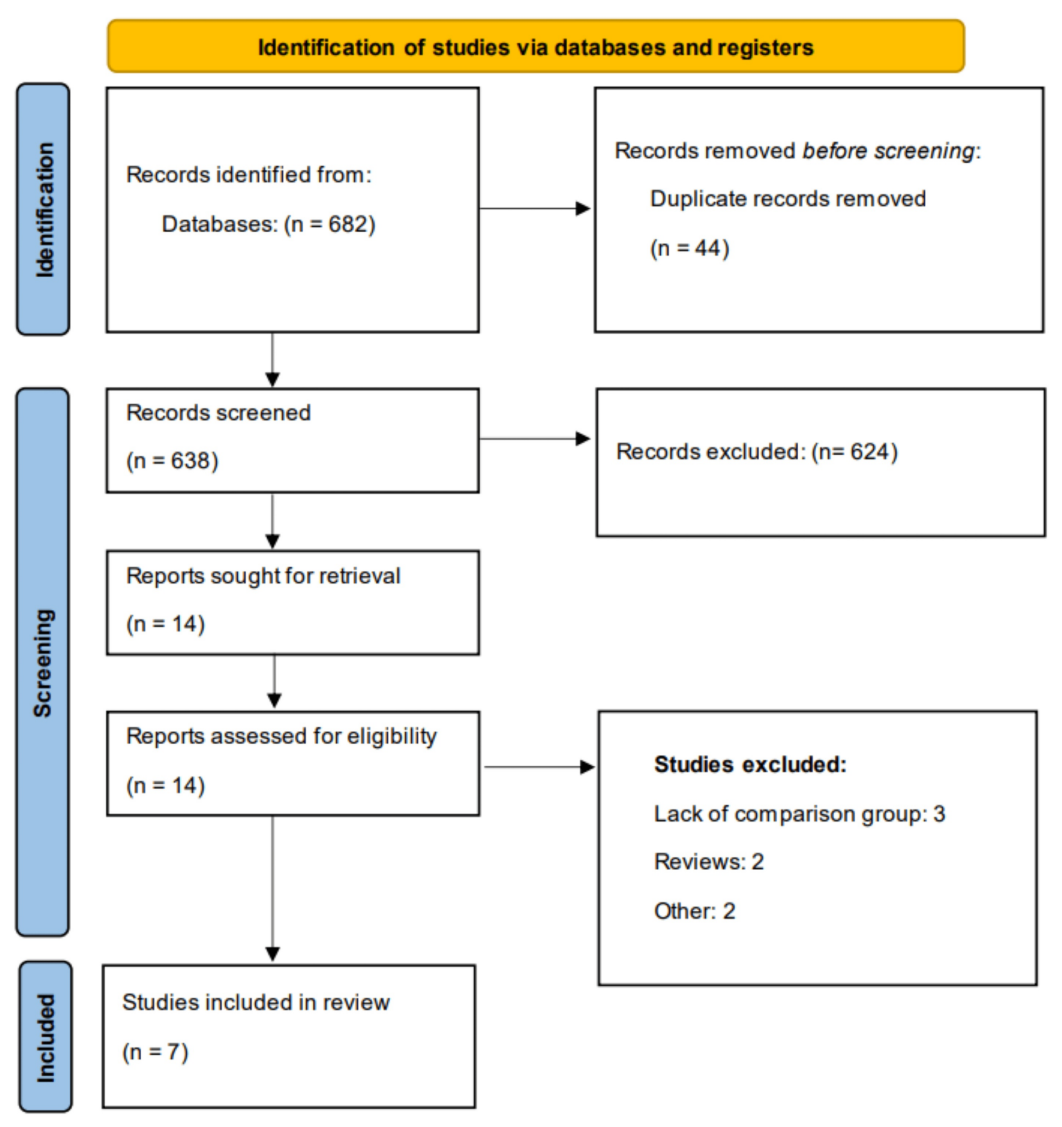
PRISMA flowchart of study selection

Through electronic database searches, a total of 682 studies were initially identified. After removing duplicates, 638 studies remained for title and abstract screening. Of these, 14 studies were assessed in full-text review, and seven met the eligibility criteria for inclusion in the meta-analysis. The study selection process is illustrated in Figure 1 (PRISMA flowchart), and the characteristics of the included studies are summarized in Table 1. The meta-analysis encompassed a pooled sample size of 5,349 participants, with a pooled prevalence of culture-negative cases estimated at 16.77%. Table 2 presents the quality assessment of the included studies. 

**Table 1:** Characteristics of included studies (n = 7) NR: not reported.

Author	Year	Region	Group	Sample size	Mean age (years)	Male (n)
Ferrera et al. [12]	2012	Spain	Positive	643	62	406
			Negative	106	61	69
Kong et al. [8]	2022	Multi-nation	Positive	2590	60.2	1808
			Negative	523	54.3	336
Lamas et al. [13]	2016	Brazil	Positive	78	41.1	50
			Negative	53	45	33
Meidrops et al. [3]	2021	Latvia	Positive	114	57.2	89
			Negative	93	53.6	61
Suardi et al. [9]	2021	Spain	Positive	918	67	613
			Negative	83	60	54
Yusuf et al. [14]	2006	United States	Positive	26	NR	22
			Negative	19		8
Zamorano et al. [15]	2001	Spain	Positive	83	52	58
			Negative	20	51	14

**Table 2:** Quality assessment of included studies Risk-of-bias assessment was conducted using the Newcastle-Ottawa Scale (NOS). Studies are awarded a maximum score of 9, with higher scores indicating better methodological quality. Studies scoring ≥7 are considered high quality, 4-6 moderate quality, and <4 low quality.

Author	Selection (4 points)	Comparability (2 points)	Outcome (3 points)	Overall quality
Ferrera et al. [12]	4	2	3	Good
Kong et al. [8]	4	2	3	Good
Lamas et al. [13]	3	2	3	Good
Meidrops et al. [3]	3	2	2	Good
Suardi et al. [9]	4	2	3	Good
Yusuf et al. [14]	3	1	2	Fair
Zamorano et al. [15]	3	1	2	Fair

Mortality 

**Figure 2:**
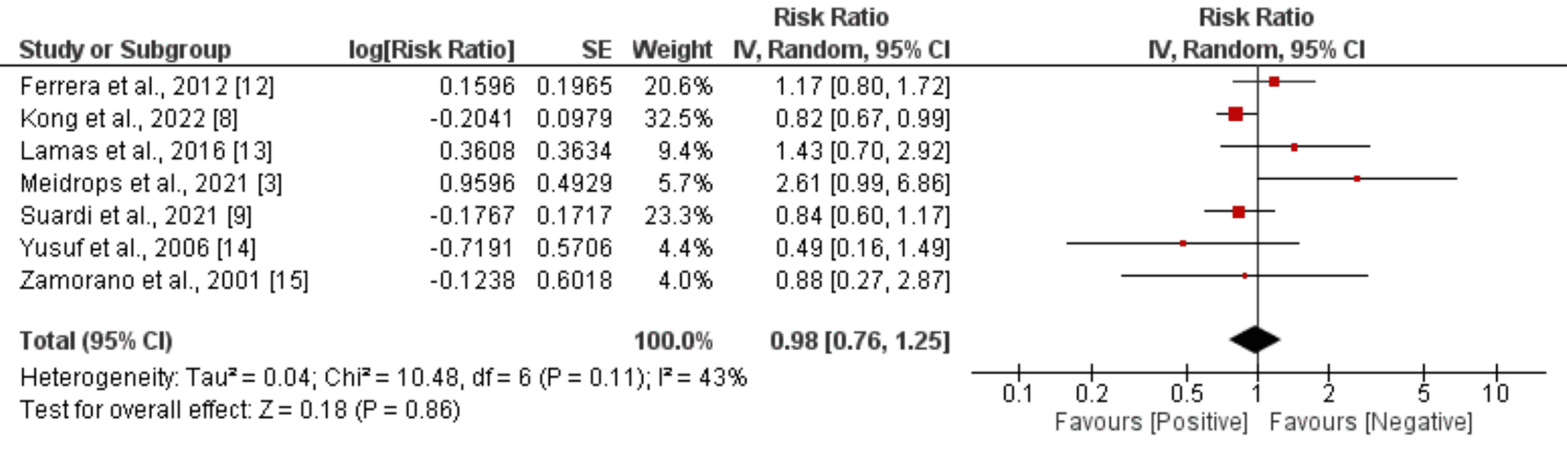
Comparison of risk of mortality between culture-positive and culture-negative endocarditis subjects References [3,8,9,12-15].

Seven studies were included to compare the death rate between culture-positive and culture-negative endocarditis subjects. As shown in Figure 2, no significant difference was found in death rate between the two groups (RR: 0.98, 95% CI: 0.76-1.25). Moderate heterogeneity was reported among the study results (I^2^: 43%). 

Embolic Events 

**Figure 3:**
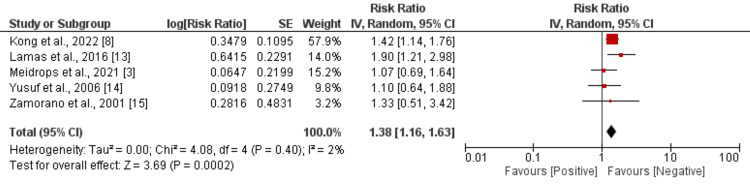
Comparison of risk of embolic events between culture-positive and culture-negative endocarditis subjects References [3,8,13-15].

Five studies were included to compare the risk of embolic events between culture-positive and culture-negative endocarditis subjects, and the results have been presented in Figure 3. Pooled analysis showed that the risk of embolic events was significantly higher in subjects with culture-positive endocarditis compared to their counterparts (RR: 1.38, 95% CI: 1.16-1.63). Low heterogeneity was reported among the study results (I^2^: 2%). 

Need for Surgery 

**Figure 4:**
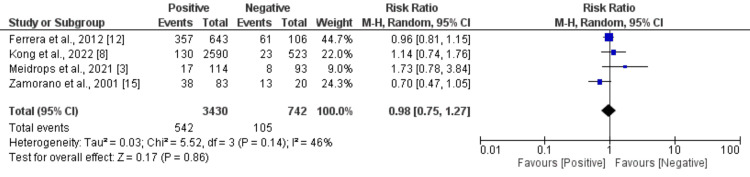
Comparison of need for surgery between culture-positive and culture-negative endocarditis subjects References [3,8,12,15].

Four studies were included to compare the need for surgery between culture-positive and culture-negative endocarditis subjects. As shown in Figure 4, no significant difference was found in the need for surgery between the two groups (RR: 0.98, 95% CI: 0.75-1.27). Insignificant heterogeneity was reported among the study results (I^2^: 46%).

Secondary Outcomes 

Table 3 presents a comparison of clinical outcomes between culture-positive and culture-negative endocarditis patients. Abscess formation was more likely in culture-positive cases, with an RR of 1.42 (95% CI: 0.94-2.14) and moderate heterogeneity (I² = 51%). Fever was also more common in culture-positive patients (RR: 1.18, 95% CI: 1.04-1.34; I² = 61%). Conversely, the risk of heart failure was lower in culture-positive cases compared to culture-negative ones (RR: 0.75, 95% CI: 0.66-0.85), with low heterogeneity (I² = 20%).

**Table 3:** Comparison of secondary outcomes *Significant at p-value <0.05. RR: risk ratio; CI: confidence interval.

Outcome	RR (95% CI)	I^2^
Abscess formation [8,9,12,13,15]	1.42 (0.94-2.14)	51%
Fever [8,9,12-15]	1.18 (1.04-1.34)*	61%
Heart failure [8,9,12,13,15]	0.75 (0.66-0.85)*	20%

Comparison of Characteristics Between Two Groups 

Table 4 compares key characteristics between culture-positive and culture-negative endocarditis patients. Mean age was higher in culture-positive subjects (mean difference: 2.99, 95% CI: 0.04-5.94), though heterogeneity was substantial (I² = 72%). Male patients were slightly more prevalent in the culture-positive group (OR: 1.32, 95% CI: 0.95-1.85), with moderate heterogeneity (I² = 52%). Notably, diabetes was significantly more common among culture-positive patients (OR: 1.43, 95% CI: 1.17-1.73), with no observed heterogeneity (I² = 0%).

**Table 4:** Comparison of characteristics ^Presented as mean difference (95% CI). *Significant at p-value <0.05. OR: odds ratio; CI: confidence interval.

Characteristics	OR (95% CI)	I^2^
Age^ [3,8,9,12,13,15]	2.99 (0.04-5.94)*	72%
Male [3,8,12-15]	1.32 (0.95-1.85)	52%
Diabetes [3,8,9,12-14]	1.43 (1.17-1.73)*	0%

Discussion 

This systematic review and meta-analysis aimed to compare clinical outcomes between patients with culture-positive and culture-negative IE. The pooled prevalence of culture-negative IE was estimated at 16.8%. To the best of our knowledge, this is the first meta-analysis to systematically compare the outcomes between these two groups. Our findings revealed no significant difference in the risk of mortality or the need for surgery between culture-positive and culture-negative IE patients. However, patients with culture-positive IE had a significantly higher risk of complications, including embolic events, compared to those with culture-negative IE. Additionally, pooled analysis showed that the risk of heart failure was significantly lower in patients with culture-positive IE. 

The present study did not show any significant difference in mortality between culture-positive and culture-negative IE subjects. None of the included studies showed any significant difference between culture-positive and culture-negative IE subjects. Several factors may explain why pooled analysis, as well as included studies, do not find significant mortality differences. Advances in empirical broad-spectrum antibiotic regimens and improved imaging techniques have enhanced early diagnosis and management, potentially mitigating the disadvantages traditionally associated with CNIE [1]. Additionally, host factors, such as comorbidities, presence of prosthetic valves, and the severity of heart failure, often play a more decisive role in determining outcomes than microbiological status alone [16]. Thus, while culture-negative status can complicate management, modern diagnostic and therapeutic strategies may help equalize mortality risks between culture-positive and culture-negative IE in contemporary practice. 

The present study also reported a higher incidence of abscess and embolic events. The higher embolic rate and abscess in culture-positive infective endocarditis (CPIE) can be attributed to several interconnected biological and pathophysiological mechanisms. First, CPIE patients harbored identifiable, actively replicating pathogens with higher virulence potential, particularly organisms like *Staphylococcus aureus* that produce proteases, toxins, and biofilm structures that inherently destabilize vegetation architecture [17]. These virulent bacteria create a more intense systemic inflammatory response, as evidenced by increased erythrocyte sedimentation rate (ESR), neutrophilia, and reduced hemoglobin levels in blood culture-positive patients, leading to enhanced cytokine release and complement activation that further compromises vegetation stability [17]. Kong et al. observed distinct embolic patterns in CPIE patients, with more peripheral and septic emboli, spondylitis, and multiple organ system involvement - complications that are characteristic of infectious rather than non-infectious embolic events. This finding underscores the fundamental biological difference between active infectious endocarditis and culture-negative disease, emphasizing that the presence of identifiable, virulent pathogens creates an inherently pro-embolic environment that requires immediate targeted antibiotic therapy and close embolic surveillance to minimize complications [8]. 

The most common cause of negative blood cultures is prior antibiotic use [18], which can lower bacterial detection rates by approximately 35% to 40% [17]. In one study, this led to a decline in positive blood culture results from 97% to 91% [7]. Pesanti and Smith [19] found that patients with negative cultures were twice as likely to have received antibiotics compared to those with positive cultures (62% versus 31%). Similarly, another investigation showed that only 64% of patients who had been given antibiotics before blood sample collection had positive cultures, compared to a 100% positivity rate among those who had not received any treatment [20]. 

With ongoing and future advancements in clinical diagnostic laboratories, combined with enhanced antimicrobial stewardship, it is plausible that the incidence of BCNE will continue to decrease. A systematic review on the epidemiology of IE published a decade ago had already suggested this possibility, noting that improvements in laboratory methods for pathogen detection might have contributed to a significant reduction in BCNE rates over time (p < 0.001) [21]. More up-to-date national data from Germany [22] and Denmark [23] have similarly shown a downward trend in BCNE prevalence among IE patients. Consequently, as molecular diagnostic tools, such as metagenomic sequencing, become more accessible and cost-effective, earlier and more accurate diagnosis of BCNE may be achievable, potentially leading to more timely and targeted treatments. 

Given the higher risk of embolic and abscess complications observed in CPIE, clinical practice should emphasize vigilant monitoring and early intervention for these patients [24]. Regular imaging, such as transesophageal echocardiography, and, when indicated, advanced modalities like PET-CT should be considered to promptly identify embolic events and peri-annular abscesses [25]. Multidisciplinary management, involving infectious disease specialists, cardiologists, and cardiac surgeons, is recommended to ensure comprehensive care and timely surgical consultation when complications arise. For all patients with IE, regardless of culture status, clinicians should maintain a high index of suspicion for new embolic phenomena and heart failure and adjust management plans accordingly. Early recognition and targeted intervention in high-risk CPIE cases may help reduce the incidence of severe complications and improve overall outcomes. 

This meta-analysis has several important limitations that must be acknowledged. The included studies exhibited significant heterogeneity in study design, ranging from prospective registry data to retrospective single-center analyses, which may have introduced varying degrees of selection bias. Diagnostic criteria for culture-negative endocarditis varied between studies, with some employing original Duke criteria while others used modified versions, potentially leading to misclassification bias. Geographic and temporal variations across studies, spanning different healthcare systems and decades, may have influenced both prevalence and outcome patterns as diagnostic capabilities evolved. The definition and detection methods for embolic events were not standardized, with some studies relying on clinical diagnosis while others used systematic imaging protocols. Microbiological diagnostic techniques varied substantially, with newer molecular methods available in some centers but not others, potentially artificially inflating culture-negative rates. Several studies focused exclusively on surgical patients, introducing selection bias toward severe cases and limiting generalizability to medically managed patients. 

## Conclusions

This systematic review and meta-analysis represents the first comprehensive comparison of clinical outcomes between blood culture-positive and blood culture-negative IE patients. Our findings demonstrate that while mortality rates and surgical intervention needs are comparable between groups, blood culture-positive patients face significantly higher risks of embolic complications. These results challenge the traditional assumption that culture-negative endocarditis necessarily portends worse outcomes. The higher embolic risk in culture-positive cases likely reflects the presence of virulent, actively replicating pathogens that destabilize vegetation architecture. Clinically, these findings emphasize the need for enhanced embolic surveillance and early intervention strategies in culture-positive patients. As molecular diagnostic techniques continue to advance, the landscape of culture-negative endocarditis may evolve, potentially improving targeted treatment approaches and overall patient outcomes in this challenging clinical scenario.
